# Co-Operation and Inter-Communication

**Published:** 1911-10-07

**Authors:** 


### CO-OPERATION AND INTER-COMMUNICATION.

### How to Make National Insurance Effective.

The discussion which took place at the largely attended Hospital Conference at Manchester, and the resolution unanimously carried, indicate the widespread opinion of voluntary hospital managers that Mr. Lloyd George, unless he improves his Bill as it stands to-day, may go down to history as the destroyer of our great system of voluntary hospitals, which have become so admirably efficient as to cause them to be universally acknowledged as the highest type of hospital efficiency yet attained in any part of the world. To say this is to give courage to hospital managers, for it passes all belief that any man in the responsible position of Chancellor of the Exchequer would put his name to legislation, the ultimate effect of which must be to cause an increase in the rating of 8s. 6½d. per head of the population each year. Such an increase in the rates would represent more than 25 per cent. of the total revenue at present raised, according to the latest returns, from local rates, including poor law, county and municipal authorities, sanitary authorities, and elementary education authorities. We do not believe that any Chancellor of the Exchequer, or any Government who supported him, could continue in office who proposed to add ten to fifteen millions a year to our national expenditure, and to present the nation in return with a guarantee that he would absolutely destroy the voluntary hospitals of this country. Yet anyone who studies the figures, and takes the trouble to go thoroughly into the sources of revenue of the voluntary hospitals as a whole, or of any important individual hospital situated in the United Kingdom, will speedily become aware that unless the National Insurance Bill provides for the administering of hospital benefit to the satisfaction of the Insurance Commissioners, as Clause 15 provides for the administration of sanatorium benefit, the Bill, when passed, must break down in practice, whilst it will certainly cripple, if it does not even destroy, a large proportion of our voluntary hospitals as they exist to-day. With the object of making this point clear, we refer the reader to the following figures, which are taken from the audited figures of 178 principal voluntary hospitals in Great Britain and Ireland. Careful inquiry shows that the probable reduction in income, under the Bill as drafted, to the various voluntary hospitals may be ultimately:—In London 23 per cent., the Provinces 47½ per cent., Scotland 45 per cent., and Ireland 29 per cent. These estimates are based upon the knowledge that the percentage of revenue of the voluntary hospitals which is secured is as follows:—

| | | |
|---|---|---|
| London | 62% | from invested property, King's Fund, patients' payments, training and employment of nurses. |
| The Provinces | 32% | from invested property, &c. |
| Scotland | 38% | do. do, |
| Ireland | 61½% | from invested property, King's Fund, patients' payments, training and employment of nurses, and grants by local authorities. |

The difference between the sum of the two percentages here given and 100 represents the estimated average receipts from casual and minor items, which constitutes a constantly varying item.

The increase in the number of people seeking hospital treatment as in-patients may be indicated by the fact that it has been stated that a careful estimate shows that the number of insured persons under Mr. Lloyd George's Bill who will be entitled to its benefits cannot be less than ten million. Now, many of these insured persons must in the ordinary course require hospital care. Where are they to obtain it, for no provision is made by Mr. Lloyd George for hospital benefit in his Bill? They cannot claim it from the voluntary hospitals, seeing that they will not be eligible to admission, because they are insured persons under the Bill. The voluntary hospital was founded to treat those members of the community who are able to maintain themselves and their families when well, but who cannot, out of their limited means, provide the cost of treatment when they, or members of their family, are overtaken by illness or accident.

Another point which it is well to bear in mind in this connection, is that if great care is not taken, sanatoria benefit, for the reasons we show in another article in our present issue, may lead to the expenditure of a very large sum of public money, which can only result in the ordinary course of events, within probably twenty years, in providing the nation with a lot of great and expensive buildings which are not required, and may so remain monuments to inefficient statesmanship and administration, like many unoccupied workhouses all over the country, and the famous forts which a former Prime Minister in perfect good faith caused to be erected for defensive purposes. We commend this point especially to the consideration of Mr. Lloyd George and the Government, because the inclusion of hospital benefit under Clause 15 will be a source of strength and not of weakness to the Bill, by putting a brake upon excessive expenditure on sanatoria in the early days of insurance. For all these reasons, and many others which we might urge, we are convinced that if the voluntary hospitals all over the country arouse themselves, organise their forces, and act together, they will render a national service by saving the voluntary system from disaster, and so arousing public opinion by seising the people of the facts that our whole system of medical relief and health provision must be immeasurably improved, to the saving of the national exchequer and the benefit of every inhabitant of these islands.

### Organise and Amend the Bill.

The first step to take is to organise public opinion; for every important hospital throughout the country to put itself at the head of an organisation within the district which it serves, with which shall be associated representatives of all the voluntary hospitals working within the particular area. This system has been adopted in several of the largest cities of the United States, and has resulted in making the hospital and health organisation and administration wonderfully efficient. We beg to repeat what was said in the discussion at Manchester, that the chief hospital chairmen should take steps to summon a meeting of the representatives of all the voluntary hospitals within his district without delay to organise hospital opinion and concert measures to protect the voluntary hospitals from the dangers with which they are threatened by Mr. Lloyd George's Bill as it stands. Each meeting should appoint a committee with power to seek an interview with the members of Parliament for the district, and to urge upon them the importance of at once bringing to the notice of the Chancellor of the Exchequer the absolute necessity that he should make certain changes in the National Insurance Bill as it stands. Such committees should not rest until they have obtained an undertaking from their M.P.s that they will stand by the voluntary hospitals and see them safeguarded before the Bill becomes law.

And here we may say one word of caution, for on the last day that the Bill was before the House, Mr. McKenna, who had charge of the Insurance Bill, resisted the amendment of Clause 15 so as to make it include hospital benefit, on the ground that the question of assistance to hospitals would be better discussed on Clause 47. Now, Clause 47 cannot, under any circumstances, be made effective if we are to safeguard the voluntary hospitals and resist effectually their ultimate absorption by the State. Clause 47 deals with the erection of sanatoria, and the only amendment to that clause which could affect hospitals would be one empowering the Government to make loans at a low rate of interest to voluntary hospitals for the purpose of erecting new pavilions on a system which should provide for the repayment of such loans, with the interest, by a sinking fund which must be provided out of the payments which would be received by the hospitals for the hospital treatment they provide for the patients who use them. We have no objection whatever to the amendment of Clause 47 to this extent, especially as it would enable the whole hospital system of the country to be consolidated, with the result that adequate hospital provision might then be forthcoming for all classes of the community, whilst the expenditure in the future upon hospitals other than voluntary hospitals by the Government must be materially decreased.

Our immediate concern is to provide the managers of every voluntary hospital with the few amendments which will effectively protect the voluntary hospitals, and materially improve the efficiency of Mr. Lloyd George's scheme of national insurance. The most material amendments are those which it is essential to get the Government to make to Clause 15, although that clause has already passed through Committee. The amendments are as follows:—

Clause 15 as amended in Committee to be further amended as follows: Clause 15 (1), line 1, for the word "sanatorium" substitute "institutional." Line 5 (*a*), delete the words "such" and "as aforesaid"; after the word "disease" insert the words "or injuries," and after the word "sanatoria" insert the word "hospitals." Line 7 (*a*), after the word "sanatoria" insert the word "hospitals." Line 9 (*b*), after the word "sanatoria" insert the word "hospitals." Line 15 (2), for the word "sanatorium" substitute the word "institutional." Line 29 (4), after the word "sanatorium" insert the words "or hospital." Clause 17, lines 2 and 3 of clause, delete the words "to hospitals and other charitable institutions, or." Clause 47 to be amended in the sense we have already set forth above.

Another amendment to which we attach importance, is that the powers taken under Clause 17, by which an approved society may subscribe, if it thinks fit, to hospitals and other charitable institutions, should be deleted. The voluntary hospitals have had enough experience of these subscriptions to know that they are the source of loss, and not a source of strength to voluntary hospitals. When such a society gives a subscription of £20 or £50 a year, its members, who may number hundreds, consider that every one of them is entitled to hospital benefit, and should receive it free of cost whenever it is claimed. Under a modified and strengthened voluntary system free relief would be confined entirely to that class of patient for whom these hospitals were originally founded, and all other patients, whether they are sent by the State or local authorities, will be paid for pro rata under the plan given later on.

To sum up, we earnestly ask the chairman of every voluntary hospital of importance throughout the United Kingdom to summon a meeting of the hospitals in his district, to appoint a small committee to represent them, and to take immediate steps to urge their representatives in Parliament to procure the amendments to the National Insurance Bill which we have just indicated. This is the first step to make this Bill really effective for good. Whenever action is taken by the hospitals in a district a notification should be sent at once to Dr. Mackintosh, M.V.O., honorary secretary of the British Hospitals Association, Western Infirmary, Glasgow, so that the whole movement may be focussed, and a great and representative deputation may be rapidly formed to interview the Chancellor of the Exchequer, should occasion arise.

### What Organisation Should Accomplish.

We have asked, Why not make the National Insurance Bill effective? How can this be done? When once the district committees representing voluntary hospitals are formed throughout the United Kingdom, they can become the basis of an organisation which should include all the agencies which at present take any part in the relief of sickness or accident or the care of dependents, or are charged with the maintenance of public health. In this way it may be possible to gather together information, and to secure by inter-communication the co-operation of every means now existing, whether it be voluntary, or municipal, or State, which deals with these important matters. Once this step is taken the complete organisation of a perfect, intelligent and thorough system of medical and health provision should speedily cease to be a dream by becoming a demonstrable fact.

